# Genetic evidence links hyperthyroidism to knee osteoarthritis

**DOI:** 10.1007/s42000-025-00648-0

**Published:** 2025-04-04

**Authors:** Tianli Xu, Limin Shen, Xiaojun Cao, Jincheng Song, Mengjie Tang, Chaoyan Yue

**Affiliations:** 1 Department of Orthopedics, The Affiliated Zhangjiagang Hospital of Soochow University, Suzhou, 215123 Jiangsu China; 2 Department of Endocrinology, The Affiliated Zhangjiagang Hospital of Soochow University, Suzhou, 215123 Jiangsu China; 3 Obstetrics and Gynecology Hospital of Fudan University, Shanghai, China

**Keywords:** Hyperthyroidism, Knee osteoarthritis, Genome-wide association study, Mendelian randomization

## Abstract

**Purpose:**

The causal relationship between hyperthyroidism and knee osteoarthritis (KOA) remains to date unknown. We aimed to examine the potential causal relationship between hyperthyroidism status and the risk of developing KOA via a bidirectional two-sample Mendelian randomization (MR) approach.

**Methods:**

Single-nucleotide polymorphism (SNP) data related to hyperthyroidism and KOA were obtained from a genome-wide association study (GWAS) in Europe. KOA was used as the outcome variable and hyperthyroidism was used as the exposure factor. The inverse-variance weighted (IVW) method served as the primary analytic tool and heterogeneity and pleiotropy were evaluated via sensitivity analysis.

**Results:**

The IVW method indicated that hyperthyroidism status has a causative influence on the risk of developing KOA [OR, 1.046; 95% confidence interval (CI), 1.013–1.080; *P* = 0.006]. No significant reverse causality was detected. Sensitivity analyses validated the robustness of these findings.

**Conclusions:**

Hyperthyroidism status can causally increase the risk of developing KOA. This result indicated that the risk of developing KOA may be decreased by controlling hyperthyroidism.

## Introduction

Knee osteoarthritis (KOA) is one of the most prevalent knee joint degenerative disorders, causing progressive pain and chronic disability [1]. Anatomical abnormalities, ageing, obesity, infection, trauma, and metabolic disorders are key risk factors for KOA [2, 3]. A systematic analysis of the Global Burden of Disease Study revealed that over 303 million people worldwide suffer from limited mobility due to OA and over 260 million of those patients with OA have KOA [4]. Changes in joint tissue structure and inflammation within the infrapatellar fat pad beneath the patella are considered to be the pathological mechanisms of KOA [1, 5]. Recently, thyroid hormone (TH) metabolism was reported to be involved in articular cartilage maintenance and the pathogenesis of KOA. THs increase the secretion of calcitonin and maintain the balance of calcium and bone metabolism, and abnormal secretion of these hormones could promote the development of KOA [6, 7].

Hyperthyroidism is a common endocrine disorder caused by thyroid synthesis and the secretion of excessive THs, impacting a series of systems such as the muscular, skeletal, cardiovascular, endocrine, and digestive systems [8]. In previous studies, investigators have reported the prevalence of hyperthyroidism to range from 0.2 to 1.3% [9, 10]. Moreover, it was suggested that hyperthyroidism status is associated with the incidence of KOA [11]. However, population-based cohort studies have shown that there is no evidence of a relationship between hyperthyroidism status as indicated by TSH and the risk of undergoing knee replacement or developing KOA [12, 13]. Additionally, there is no direct evidence supporting the theory that hyperthyroidism status directly influences the development of KOA or vice versa. Therefore, a new method is needed to firmly establish the causality of the association between hyperthyroidism status and the risk of developing KOA.

Although randomized controlled trials (RCTs) represent the highest level of evidence among all studies, RCTs are complex and expensive. Mendelian randomization (MR) has now been developed as a novel and powerful analytical method that uses genetic variants as instrumental variables (IVs) to assess the causal relationship between exposure and clinical disease [14, 15]. MR can be used to avoid the reverse causality of risk factors and outcomes, as genotypes are randomly assigned at fertilization. MR has been used to analyze causal relationships in various contexts, including the associations of sarcopenia-related traits [16], iron status [17], ADAMTS5 [18], increased glycated hemoglobin (HbA1c) [19], body mass index (BMI), bone mineral density (BMD) [20], osteoporosis [21], metabolites and metabolic pathways [22], and thyroid dysfunction [11] with the risk of developing KOA. However, the causal relationship between hyperthyroidism status and the risk of developing KOA has not been explored via MR analysis. On the basis of the detailed phenotype of conditions from large-scale genome-wide association studies (GWASs), in the present study, two-sample MR was used to investigate the causal correlation between hyperthyroidism status and the risk of developing KOA.

## Methods

### Study design

**Fig. 1:**
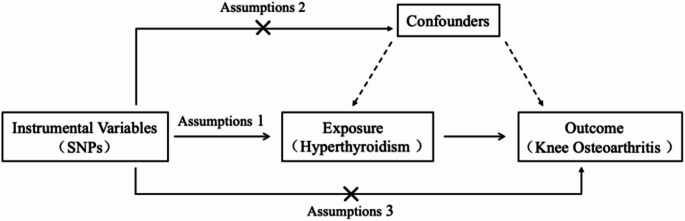
The schematic representation of this study and the three assumptions of Mendelian randomization analysis: (1) The selected IVs are closely related to hyperthyroidism. (2) The used genetic IVs are independent of the currently known confounding factors. (3) The selected SNPs have no direct influence on the outcome of knee osteoarthritis, but only influence the outcome through hyperthyroidism

As shown in Fig. 1, we used a two-sample MR design to analyze the causal effect of hyperthyroidism on KOA. Three fundamental assumptions were used, as follows: (1) the selected genetic variants are directly related to hyperthyroidism; (2) the selected genetic variants are unrelated to any other confounders; and (3) the IVs used influence KOA solely through hyperthyroidism. Summary single-nucleotide polymorphisms (SNPs) for hyperthyroidism and KOA were obtained from GWAS datasets separately. The GWAS database is an open-access data infrastructure and all studies obtained institutional review board approval; thus, additional ethics approval was unnecessary. In order to investigate the genetic evidence linking hyperthyroidism to KOA, the current study employed two-sample MR.

### Data sources

The summary datasets of hyperthyroidism were obtained from the GWAS datasets of 460,499 (3577 patients with hyperthyroidism and 456,942 controls) European individuals (Table 1). The raw data can be found in the study of Sakaue et al. []. The GWAS data for KOA were derived from the UK biobank, including 24,955 KOA cases and 378,169 controls (the raw data were available in the study by Saori Sakaue et al.) [24]. The participants were all from Europe.

**Table 1:** Summary of the GWAS included in this MR study

Exposure/Outcomes	GWAS ID	Sample size	Number of SNPs	Sex	Population	Website
Hyperthyroidism	ebi-a-GCST90018860	460 499	24 189 279	NA	European	https://gwas.mrcieu.ac.uk/datasets/ebi-a-GCST90018860/
Knee Osteoarthritis	ebi-a-GCST007090	403 124	29 999 696	NA	European	https://gwas.mrcieu.ac.uk/datasets/ebi-a-GCST007090/

*GWAS* genome-wide association studies, *SNP* single-nucleotide polymorphism

### IV selection criteria

All 14 selected SNPs were directly and significantly related to the risk of developing hyperthyroidism on the basis of a stringent significance threshold (*p* < 5 × 10^− 7^) and independence (*r*^*2*^ < 0.001, *kb* = 10000). Detailed information concerning the 14 SNPs is provided in Table 2. We employed the F statistic, calculated as F = (N-2) *R^2/(1-R^2), to assess weak instrument bias. An F value greater than 10 implies that the instrument strongly represents the risk of developing hyperthyroidism.

**Table 2:** Characteristics of SNPs from GWAS

SNP	Effect-allele. exposure	Other allele. exposure	Hyperthyroidism				Knee Osteoarthritis			F
			β	SE	*P*		β	SE	*P*	
rs1023586	C	T	0.248	0.023	3.77 × 10^− 28^		0.030	0.010	0.002	120.71
rs10917469	G	A	0.160	0.031	1.83 × 10^− 7^		0.010	0.013	0.417	27.23
rs12274645	A	G	0.182	0.036	3.81 × 10^− 7^		0.016	0.015	0.287	61.36
rs2476601	G	A	-0.296	0.038	4.78 × 10^− 15^		-0.008	0.015	0.585	42.31
rs28808421	G	A	0.374	0.058	8.05 × 10^− 11^		0.006	0.019	0.763	82.78
rs3087243	A	G	-0.204	0.022	7.94 × 10^− 20^		-0.019	0.009	0.040	194.78
rs3134971	C	A	0.546	0.039	2.29 × 10^− 44^		0.042	0.014	0.003	48.79
rs4338740	C	T	0.184	0.026	2.87 × 10^− 12^		-0.006	0.011	0.606	30.48
rs58722186	T	C	0.136	0.023	5.23 × 10^− 9^		-0.004	0.010	0.723	30.37
rs604912	G	A	0.120	0.022	3.15 × 10^− 8^		-0.009	0.010	0.322	47.41
rs6131010	G	A	0.131	0.024	3.60 × 10^− 8^		-0.007	0.011	0.526	26.03
rs72498347	T	C	-0.331	0.048	6.00 × 10^− 12^		0.0128	0.026	0.626	29.03
rs75024498	A	G	0.220	0.043	3.28 × 10^− 7^		-0.005	0.018	0.796	120.71
rs75478527	G	A	0.351	0.065	7.19 × 10^− 8^		-0.020	0.024	0.424	27.23

*SNP* single-nucleotide polymorphism; *β* per allele effect on the exposure; *SE* standard error; *P p*-value for the genetic association; *F* statistic, calculated as F = (N-2) *R^2/(1-R^2)

### MR analysis

In this MR analysis, we conducted various MR methods to evaluate the causal relationship between hyperthyroidism status and the risk of developing KOA. The etiological effects of hyperthyroidism status on the risk of developing KOA were analyzed via inverse-variance weighted (IVW) [25], MR-Egger regression (MR-Egger), weighted median (WME), and weighted mode. IVW yields accurate estimates when all SNPs satisfy the assumption of being a valid tool variable [26]. WME provides robust estimates of outcomes even when nearly 50% of genetic variants are invalid [27]. MR-Egger and the MR pleiotropy residual sum and outlier (MR-PRESSO) test were used to detect broad horizontal pleiotropy to ensure that IVs satisfied the assumptions. As the MR-Egger method could be affected by abnormal genetic variation, a leave-one-out analysis was performed to avoid horizontal pleiotropy caused by a single SNP. Cochran’s Q test was conducted to evaluate the statistical heterogeneity of single SNPs via the IVW method, with *p* < 0.05 considered to indicate significant heterogeneity. The Steiger directionality test was used to identify the correct causal direction between the outcome and exposure [28]. Funnel plots in which each SNP had a single Wald ratio revealed the directional horizontal pleiotropy of the IV. The occurrence of false positives was reduced using the threshold of the *p* value corrected by the Bonferroni method (*p* < 0.016). We used the “TwoSampleMR” and “MR-PRESSO” packages in R version 4.3.0 for analysis.

## Results

**Fig. 2:**
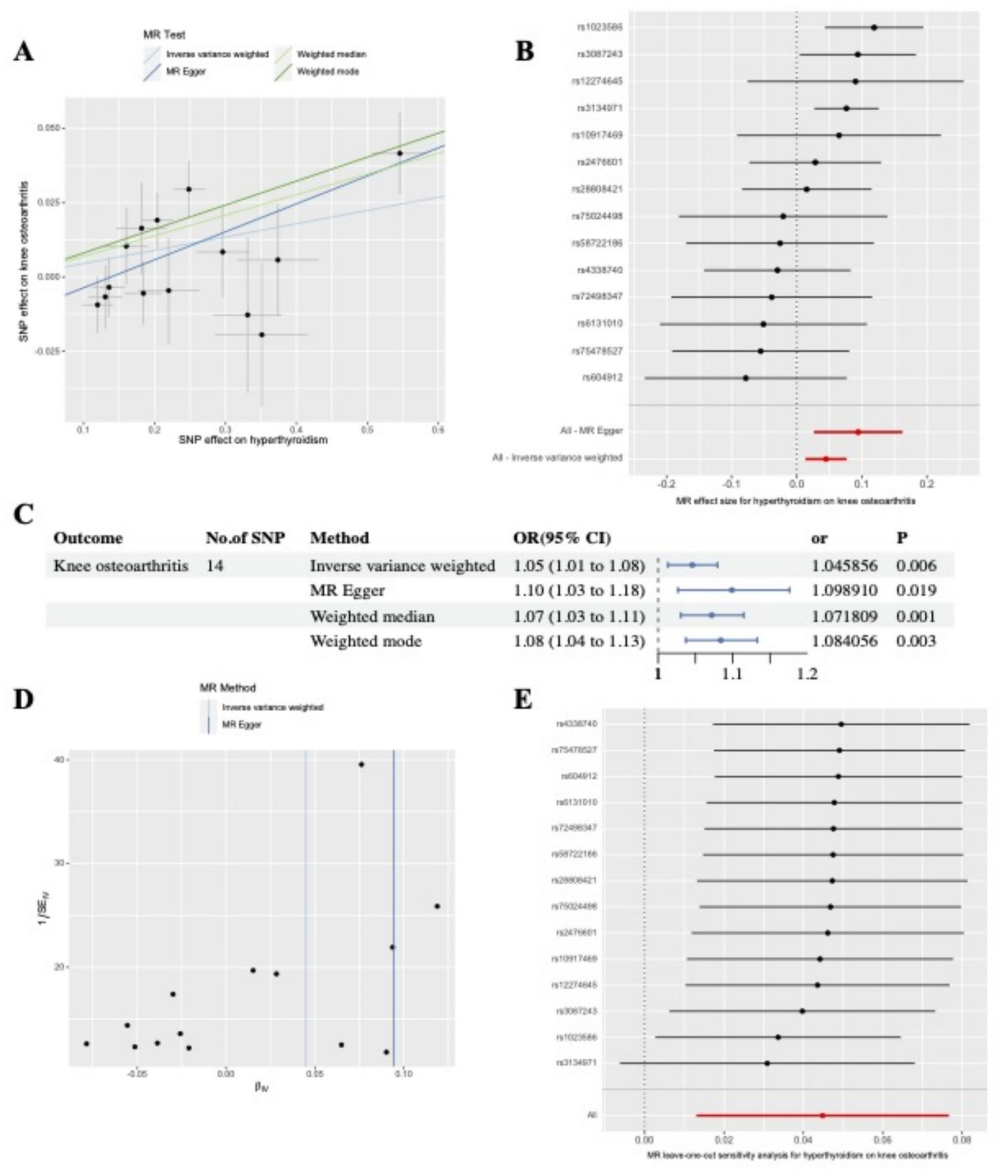
Causal relationship of hyperthyroidism on knee osteoarthritis. **A**, Scatter plot of the causal effect of hyperthyroidism on the risk of knee osteoarthritis. The slope of each line represents the causal estimate. **B**, Forest plot of the causal effect of each SNP related to hyperthyroidism on knee osteoarthritis. The red line represents the results of MR-Egger and IVW. **C**, Causal effect estimates of hyperthyroidism on knee osteoarthritis using four MR methods. The circles represent the point estimates of the causal effects, and the error bars represent the 95% CIs. **D**, Funnel plots for the overall heterogeneity of the effect of hyperthyroidism on the risk of knee osteoarthritis **E**, Leave-one-out plot of the causal effect of hyperthyroidism on the risk of knee osteoarthritis when removing SNPs one by one. Beta, the effect size of the exposure on outcome

The MR estimates of the different approaches we used to evaluate the causal associations between hyperthyroidism status and the risk of developing KOA are shown in Fig. 2C; Table 3, indicating that hyperthyroidism status was causally associated with an increased risk of developing KOA (*OR*, 1.046; *95% CI*, 1.013–1.080; *p* = 0.006). Those results were confirmed via WME (*OR*, 1.072; *95% CI*, 1.030–1.115; *p* = 0.030), MR‒Egger (*OR*, 1.099; *95% CI*, 1.027–1.176; *P* = 0.026) and weighted mode (*OR*, 1.084; *95% CI*, 1.037–1.133; *p* = 0.037). The correlation between hyperthyroidism status and the risk of developing KOA is illustrated in the scatter plot in Fig. 2A, representing the causative effect of each SNP on the risk of developing KOA. A forest plot of the causal effect of each SNP related to hyperthyroidism on KOA is displayed in Fig. 2B. The results of the funnel plot and MR-Egger intercept analysis indicated the absence of pleiotropy (Table 3; Fig. 2D, *p* > 0.05). The same results were detected in the MR-PRESSO-corrected data *(p* = 0.166). No positive results were found in the heterogeneity analysis (Cochran’s Q test was greater than 0.05) (Table 3). The leave-one-out test revealed no problematic SNPs, as shown in Fig. 2E. Our analysis revealed no reverse causality in the Steiger test (*p* < 0.05). On the basis of the above results, we believe that genetically predicted hyperthyroidism status is a risk factor for the development of KOA.

**Table 3:** Association of hyperthyroidism with knee osteoarthritis in sensitivity analysis

Method	OR (95% CI)	*P*	Cochrane Q (*P*)	Intercept in MR-Egger regression (*P*)
Inverse variance weighted	1.046 (1.013–1.080)	0.006	17.42 (0.181)	
MR Egger	1.099 (1.027–1.176)	0.026	14.40 (0.276)	-0.013 (0.138)
Weighted median	1.072 (1.030–1.115)	0.030		
Weighted mode	1.084 (1.037–1.133)	0.037		

*CI* confidence interval, *MR* Mendelian randomization, *OR* odds ratio

## Discussion

On the basis of the genetic data obtained from the GWAS datasets, a bidirectional two-sample MR analysis was conducted to explore the causal relationship between hyperthyroidism status and the risk of developing KOA. We demonstrated a possible strong causal relationship between hyperthyroidism status and the risk of developing KOA. Genetic susceptibility to hyperthyroidism is associated with increased KOA prevalence.

Hyperthyroidism is characterized by suppressed TSH levels and high fT3 and/or fT4 levels, along with an increased basal metabolic rate (BMR) [29]. A NHANES study that included 8478 subjects to analyze the associations between TH indices and OA revealed that higher levels of fT3/fT4 and lower levels of TSH were related to an increased prevalence of OA, including that of KOA [7]. Thyroid disorders significantly affect the skeletal system and the maintenance of appropriate bone development and strength. The conversion of thyroxin (T4) hormones inside cells to triiodothyronine (T3) depends on 2 iodothyronine deiodinase (DOI2) activity and subsequently plays an important role in the development of fetal bone [30]. Inside the cell, T3 and T4 interact with nuclear TH receptors (TRs). TRα1 and TRβ1 are widely distributed in undifferentiated and proliferating chondrocytes [31]. T3 also stimulates the expression of matrix metalloproteinase-13 (MMP13) to promote cartilage mineralization and degradation [32, 33]. A previous study of 109 patients with thyroid diseases revealed that both hypothyroid and hyperthyroid patients had increased joint effusion, which was associated with the incidence of KOA [11]. In contrast to most previous studies, Hellevik et al. reported that no significant relationship was found between hypothyroidism or hyperthyroidism status and the risk of undergoing knee joint replacement due to KOA [34]. Our MR analysis results revealed that, unlike in previous controversial studies, there was a unidirectional causal relationship between hyperthyroidism status and the risk of developing KOA, indicating that hyperthyroidism status is a risk factor for the development of KOA.

KOA involves local and systemic low-grade inflammation accompanied by synovial hyperplasia and low-grade inflammation in the synovial intima [35]. KOA is a complex process influenced by age, sex, increased HbA1c, the BMR, sarcopenia, obesity, and joint wear and tear [16, 19, 36, 37]. Patients with hyperthyroidism always have increased BMRs, especially when they exercise regularly [29, 38]. When the BMR is high, inflammation, catabolism, and cellular reactive oxygen species are increased, leading to the progression of KOA [38–41]. Pörings et al. investigated the synovial TH network in OA patients. Cellular membrane transporters of THs, DOIs, and TRs are present in the synovial tissue of KOA patients. During low-grade inflammation, the hormone-activating DOI2 increases, and DOI3 decreases, leading to higher T3 levels [42]. Thus, the presence of DOIs could regulate local TH levels in synovial tissue and synovial fibroblasts (SFs). This finding is consistent with our findings. Our MR analysis revealed that, unlike in previous studies, hyperthyroidism status was causally related to the incidence of KOA, indicating that hyperthyroidism is the cause of KOA.

In brief, this study evaluated the causal relationship between hyperthyroidism status and the risk of developing KOA at the genetic level, providing genetic evidence to address controversies in the field. We used multiple MR methods to ensure the accuracy and validity of the results and MR-PRESSO to detect broad horizontal pleiotropy and ensure validity and reliability. The importance of hyperthyroidism status as regards the risk of developing KOA provides innovative insights for future research on the prevention of KOA.

However, our study has several limitations. First, the GWAS database included only the European population, and whether our conclusions could be replicated in non-European populations needs to be explored. Second, owing to database limitations, the latest statistical data could not be analyzed and subgroup stratification, such as by sex and age of the population, could not be performed. Third, the biological mechanism of the causal association between hyperthyroidism status and the risk of developing KOA remains unclear. Therefore, further clinical and basic studies are needed to explore the mechanisms involved in this relationship, which will considerably facilitate the devising of treatment methods.

In conclusion, our study revealed a bidirectional causal association between hyperthyroidism and KOA. On the basis of our results, it is reasonable to advise KOA patients to undergo routine screening of thyroid function. Proper management of hyperthyroidism is important for decreasing the incidence of KOA.

## Data Availability

The data underlying this article will be shared on reasonable request to the corresponding author.
